# Supplementary material for: Antimicrobial agents for the treatment of enteric fever chronic carriage: A systematic review
Source: PLoS One. 2022 Jul 29;17(7):e0272043. doi: 10.1371/journal.pone.0272043 (PMC9337697; doi:10.1371/journal.pone.0272043)
Supplement: S1 File — (PDF) [file pone.0272043.s002.pdf]

S1. Search strategy

Search using OVID SP on databases: OVID MEDLINE(R) ALL from 1946-2021 and EMBASE Classic + EMBASE from 1947-2021 AND Web of Science Core Collection 1946-2021

1 (typhoid or paratyphoid or salmonella typhi or salmonella paratyphi or enteric fever).mp. [mp=ti, ab, hw, tn, ot, dm, mf, dv, kf, fx, dq, nm, ox, px, rx, ui, sy]
2 (chronic carri* or disease carrier or carrier state or typhoid carri* or paratyphoid carri*).mp. [mp=ti, ab, hw, tn, ot, dm, mf, dv, kf, fx, dq, nm, ox, px, rx, ui, sy]
3 (antibacterial or antibiotic or antibiotic treatment or antibacterial treatment or amoxicillin or ampicillin or ciprofloxacin or quinolone or norfloxacin or chloramphenicol or co-trimoxazole or trimethoprim-sul* or septrin or antibacterial agent or antibiotic agent or penicillin or nalidixic acid or ofloxacin or sulphonamide).mp. [mp=ti, ab, hw, tn, ot, dm, mf, dv, kf, fx, dq, nm, ox, px, rx, ui, sy]
4 1 and 2 and 3
5 remove duplicates from 4
