# Supplementary material for: Antimicrobial agents for the treatment of enteric fever chronic carriage: A systematic review
Source: PLoS One. 2022 Jul 29;17(7):e0272043. doi: 10.1371/journal.pone.0272043 (PMC9337697; doi:10.1371/journal.pone.0272043)
Supplement: S1 Protocol — (PDF) [file pone.0272043.s003.pdf]

S3: Systematic Review Protocol

Title: How effective are antimicrobials in treating enteric fever chronic carriage?

Version 1.2

Date: 07/01/2020

Authors:

Dr Naina McCann

University College Hospital, London

1. Background

Enteric fever is a non-specific febrile illness caused by infection with the gram negative bacteria *Salmonella enteric* serovar Typhi (S. typhi) or *Salmonella enterica* serotype paratyphi A, B or C (S. paratyphi). It remains an important public health problem with approximately 15 million cases reported globally each year (1).

Following acute enteric fever and clinical resolution of symptoms a small proportion of patients continue to excrete *S. typhi* or *S. paratyphi* in their stool (or rarely urine). These patients are asymptomatic but pose a risk of onward transmission to others. Given S. Typhi and S. Paratyphi are human-restricted pathogens these chronic carriers are likely to play an important role in maintaining the reservoir of infection.

There is currently very limited guidance on how, when identified, these chronic carriers should be treated. Treatment options that have been investigated previously include extended courses of antimicrobials or cholecystectomy. The invasive nature of cholecystectomy in practice means this is unlikely to be a appropriate first-line option. Antimicrobials therefore tend to be the suggested treatment option where guidance does exist.

The previous WHO guidelines from 2004 suggest options of amoxicillin, co-trimoxazole or ciprofloxacin for treatment of chronic carriage (2). The evidence to support this appears to be limited and out of date. There has been no systematic review of the evidence on this topic to review the evidence surrounding antimicrobial interventions. We think this would be of vital importance, not only for clinicians in treating patients with chronic carriage, but for public health and the eventual goal of eliminating enteric fever.

2. Research question

This systematic review seeks to establish the effectiveness of a course of antimicrobials in treating enteric fever chronic carriage. It will review the different antimicrobials used to treat chronic carriage and their associated efficacy in clearing *S.* Typhi or *S.* Paratyphi from the stool.

3. Methods

3.1 Search strategy

The search strategy will access published material relating to the topic. A search strategy is outlined below:

1) Scoping searches carried out to identify existing literature on the topic
2) A limited search of MEDLINE and EMBASE will be performed to identify key words for searching
3) Terms identified in this way and synonyms will be used to perform an extensive search of the literature using MEDLINE, EMBASE and Web of Science databases
4) Reference lists and bibliographies of the articles identified from the above will be searched

Words identified from key word searching and therefore used in the final search are:
typhoid OR paratyphoid OR salmonella typhi OR salmonella paratyphi OR enteric fever AND
chronic carriage OR disease carrier OR carrier state OR typhoid carrier OR paratyphoid carrier AND
antibiotic OR antibacterial OR antibacterial treatment OR antibiotic treatment OR antibacterial agent OR antibiotic agent OR amoxicillin OR ampicillin OR penicillin OR ciprofloxacin OR quinolone OR norfloxacin OR ofloxacin OR chloramphenicol OR septrin OR co-trimoxazole OR trimethoprim-sulfamethoxazole OR sulfonamide

Articles published in the last 70 years will be searched (1946 onwards).

3.2 Inclusion criteria

Table 1 – Summary PICO Table

Population	Adults with confirmed enteric fever chronic carriage (positive stool/urine culture > 1 year)
Intervention	Treatment course of antimicrobials
Comparator	Any
Outcomes	Clearance of stool at minimum 3 months post treatment
Study design	Any interventional study
Setting	Global

3.2.1 Participants

This study will review the effect of the intervention in adult patients (>18 years) globally with enteric fever chronic carriage.

In keeping with relevant literature the term 'chronic carriage' will be defined as a positive stool (or urine) culture for *S.* Typhi or *S.* Paratyphi at *least 1 year* following acute enteric fever infection. Given that it is possible for patients to develop chronic carriage without an acute enteric fever illness studies including patients without any history of symptomatic enteric fever will be included.

Enteric fever is defined as infection with the pathogens *S*. Typhi or *S.* Paratyphi (A or B).

Patients should be asymptomatic and not have features of persistent enteric fever.

3.2.2 Intervention

This study will investigate the effect of antimicrobial treatment (a course of any antibiotic) on enteric fever chronic carriage.

There are expected variations in the intervention groups, including antimicrobial type, dose and length of course. Only antimicrobials still used in practice today will be included.

3.2.3 Outcomes

The primary outcome of interest is eradication i.e. negative stool cultures following treatment (at a minimum of 4 weeks post treatment and a minimum of 3 samples).

A secondary outcome of side effects of antimicrobial therapy will be collected and evaluated if reported consistently.

3.2.4 Types of studies

This review will include any interventional trial where antimicrobials have been given to treat chronic carriage.

Case reports and case series of under 10 cases will be excluded.

3.3 Exclusion criteria

- Children (< 18 years)
- No clear definition of how chronic carriers identified
- Convalescent carriers (i.e. patients with < 1 year of positive stool/urine cultures)
- Patients with evidence of untreated acute enteric fever e.g. ongoing fevers or bacteremia
- Use of antimicrobial no longer used in clinical practice
- Use of antimicrobial alongside another intervention e.g. surgery
- Trials with no outcome data

3.4 Quality assessment

Articles identified will be screened using the abstract and those that meet the inclusion criteria will be obtained for quality assessment.

The studies will be assessed independently for methodological validity by two reviewers prior to inclusion in the review using a validated quality assessment for the appropriate studies e.g. Newcastle-Ottawa Quality Assessment Scale. Any disagreements between the two reviewers shall be resolved by consensus. A third reviewer will be consulted if necessary.

3.5 Data extraction

Following assessment of quality papers will be grouped into study types and data extracted. A data extraction tool will be designed to extract relevant data from all papers including: name and dosing of antimicrobial therapy used, number of patients given antimicrobial treatment and number of patients given control treatment, study outcomes and length of follow-up.

3.5 Data analysis

Primary outcome looked at will be number of patients eradicated, or eradication proportion (i.e. number of those eradicated vs number of those not eradicated) for each antimicrobial. Categorical variables will be compared using Fishers exact test.

For the RCT data where possible, an odds ratio and 95% confidence interval will be calculated. If appropriate and more than 1 RCT identified a meta-analysis will be performed.

If other study types are included in the final analysis appropriate data analysis will be performed using Microsoft Excel and R Studio version 1.4.1103.

4. Time frame

12 months

5. References

1. Stanaway JD, Reiner RC, Blacker BF, Goldberg EM, Khalil IA, Troeger CE, et al. The global burden of typhoid and paratyphoid fevers: a systematic analysis for the Global Burden of Disease Study 2017. Lancet Infect Dis [Internet]. 2019 Apr 1 [cited 2021 Mar 17];19(4):369–81. Available from: http://dx.doi.org/10.1016/
2. WHO. The diagnosis, treatment and prevention of typhoid fever. World Heal Organ. 2010;
