# Supplementary material for: Antimicrobial agents for the treatment of enteric fever chronic carriage: A systematic review
Source: PLoS One. 2022 Jul 29;17(7):e0272043. doi: 10.1371/journal.pone.0272043 (PMC9337697; doi:10.1371/journal.pone.0272043)
Supplement: S2 File — (PDF) [file pone.0272043.s004.pdf]

S4. Comparing categorical variables, R file.

```
R version 4.0.3 (2020-10-10) -- "Bunny-Wunnies Freak Out"
Copyright (C) 2020 The R Foundation for Statistical Computing
Platform: x86_64-apple-darwin17.0 (64-bit)

R is free software and comes with ABSOLUTELY NO WARRANTY.
You are welcome to redistribute it under certain conditions.
Type 'license()' or 'licence()' for distribution details.

  Natural language support but running in an English locale

R is a collaborative project with many contributors.
Type 'contributors()' for more information and
'citation()' on how to cite R or R packages in publications.

Type 'demo()' for some demos, 'help()' for on-line help, or
'help.start()' for an HTML browser interface to help.
Type 'q()' to quit R.

[Workspace loaded from ~/Documents/UCH CRF/Typhoid SR/.RData]

> source('~/Documents/UCH CRF/Typhoid SR/S4. R file for comparing variables.R')
> #file for comparing categorical variables in SR
> #fisher test used for all given small numbers
>
> #comparing IV vs PO treatment of amox ?significantly different
> IV_PO <- matrix(c(0,33, 19, 49), ncol = 2, byrow = TRUE)
> colnames(IV_PO) <- c("IV", "PO")
> rownames(IV_PO) <- c("Not Eradicated", "Eradicated")
> IV_PO
               IV PO
Not Eradicated  0 33
Eradicated     19 49
> fisher.test(IV_PO)

	Fisher's Exact Test for Count Data

data:  IV_PO
p-value = 0.0002635
alternative hypothesis: true odds ratio is not equal to 1
95 percent confidence interval:
 0.0000000 0.3508122
sample estimates:
odds ratio
         0
```

```
>
> #comparing those taking amoxicillin, gallstones vs no gallstones
> Amox_gallstones <- matrix(c(13,9,16,34), ncol = 2, byrow = TRUE)
> colnames(Amox_gallstones) <- c("Gallstones", "No Gallstones")
> rownames(Amox_gallstones) <- c("Not eradicated", "Eradicated")
> Amox_gallstones
               Gallstones No Gallstones
Not eradicated         13             9
Eradicated             16            34
> fisher.test(Amox_gallstones)

	Fisher's Exact Test for Count Data

data:  Amox_gallstones
p-value = 0.03924
alternative hypothesis: true odds ratio is not equal to 1
95 percent confidence interval:
 0.9656051 9.8986009
sample estimates:
odds ratio
  3.018804

>
> #comparing those taking Fq to those taking Amox
> FQ_Amox_erad <- matrix(c(2,33,22,68), ncol = 2, byrow = TRUE)
> colnames(FQ_Amox_erad) <- c("Fq", "Amox")
> rownames(FQ_Amox_erad) <- c("Not eradicated", "eradicated")
> FQ_Amox_erad
               Fq Amox
Not eradicated  2   33
eradicated     22   68
> fisher.test(FQ_Amox_erad)

	Fisher's Exact Test for Count Data

data:  FQ_Amox_erad
p-value = 0.0212
alternative hypothesis: true odds ratio is not equal to 1
95 percent confidence interval:
 0.02038353 0.84733844
sample estimates:
odds ratio
 0.1892283

>
> #comparing those taking Fq to those taking amox (all with gallstones)
> FQ_Amox_gallstones <- matrix(c(2,13,9,16), ncol = 2, byrow = TRUE)
```

```
> colnames(FQ_Amox_gallstones) <- c("Fq", "Amox")
> rownames(FQ_Amox_gallstones) <- c("Not eradicated", "eradicated")
> FQ_Amox_gallstones
               Fq Amox
Not eradicated  2   13
eradicated      9   16
> fisher.test(FQ_Amox_gallstones)

        Fisher's Exact Test for Count Data

data:  FQ_Amox_gallstones
p-value = 0.1582
alternative hypothesis: true odds ratio is not equal to 1
95 percent confidence interval:
 0.02535116 1.72179031
sample estimates:
odds ratio
 0.2818589
```
