# Supplementary material for: Antimicrobial agents for the treatment of enteric fever chronic carriage: A systematic review
Source: PLoS One. 2022 Jul 29;17(7):e0272043. doi: 10.1371/journal.pone.0272043 (PMC9337697; doi:10.1371/journal.pone.0272043)
Supplement: Attachment — Submitted filename: Response_to_reviewers_May.docx [file pone.0272043.s005.docx]

Dr Naina McCann

Hospital of Tropical Diseases

University College London Hospital

13th May 2022

Dear Editor,

We are grateful for the opportunity to respond to the reviewers’ comments and for their suggestions which we believe have improved the manuscript.

I have provided a point-by-point response to the reviewers below:

Reviewer #1: The MS reports on antimicrobial agents for the treatment of enteric fever chronic carriage: A systematic review. In this review the author presented about the efficacy of different antimicrobials in treating enteric fever chronic carriage.   Author also states that research is needed to investigate whether these antimicrobials remain appropriate treatment options or whether alternative interventions are more effective. But, the presentation of MS is not good enough, due to this it is very hard  to understand MS.  I have some questions about MS.

1.  Author (s) should rewrite the content so the reader can understand the MS.2. It is very difficult to understand the Table-1, moreover some part of this table is missing in the MS.

Apologies, there were some formatting errors in the initial manuscript submitted and therefore some of the information in the tables were not fully visible. This has been amended and all the data in all three tables are now fully visible. We hope this has significantly improved the presentation, and therefore understanding, of the manuscript in full.

In addition, we requested a proof-read of the manuscript by an additional person, a clinical academic but not in the field of enteric fever research, to ensure the manuscript was understandable and presented well. They reviewed the manuscript and made a few small comments which have been incorporated in this revision, but commented that the overall presentation of the manuscript was good and easy to understand.

If there are further areas of the manuscript that are still hard to understand we would be happy to amend these if advised where the issues are.

Reviewer #2: This manuscript by MacCannN, Scott P, and colleagues entitled ''Antimicrobial agents for the treatment of enteric fever chronic carriage: A systematic review'' proposes to address the efficacy of different antimicrobials in treating enteric fever chronic carriage. In addition, this work shows the evidence for fluoroquinolones and amoxicillin as antimicrobial interventions for treatment of enteric fever carriage. The data collected in this systemic review could be of fundamental importance.
Some minor points to consider are included below:

In line 41: Please add comma(,) after 'identified'.
This has been added
In line 218, please correct the data 'were' synthesized.
This has been changed
Table 1, table 2 and table 3 are incomplete. Please complete them.

All three tables have been amended so all the data is fully visible.

In addition please find our response to the Journal Requirements requested:

Please ensure that your manuscript meets PLOS ONE's style requirements, including those for file naming. The PLOS ONE style templates can be found at 
<https://journals.plos.org/plosone/s/file?id=wjVg/PLOSOne_formatting_sample_main_body.pdf> and 
<https://journals.plos.org/plosone/s/file?id=ba62/PLOSOne_formatting_sample_title_authors_affiliations.pdf>

We have amended the manuscript as per the style requirements and template.

Please note that in order to use the direct billing option the corresponding author must be affiliated with the chosen institute. Please either amend your manuscript to change the affiliation or corresponding author, or email us at [plosone@plos.org](mailto:plosone@plos.org) with a request to remove this option.

University College London is listed as an chosen institute that has an Institutional Account with Plos. The corresponding author is affiliated with University College London and has a UCL email address. Please let us know if something additional is required.

We note that you have stated that you will provide repository information for your data at acceptance. Should your manuscript be accepted for publication, we will hold it until you provide the relevant accession numbers or DOIs necessary to access your data. If you wish to make changes to your Data Availability statement, please describe these changes in your cover letter and we will update your Data Availability statement to reflect the information you provide.

All data is included within the manuscript itself therefore respository information for the data is not required. This has been amended in the submission.

Request for manuscript to be converted to LaTeX format.

Unfortunately we offer apologies but we have been unable to successfully convert the manuscript from a doc to LaTeX format. Being unfamiliar with LaTeX it has taken us a significant number of hours to learn the basics of LaTex and convert the file from a word file into a .tex file in the correct format. Although able to correctly convert all the text (which we would be happy to upload and send) we have been unable to convert the tables and bibliography successfully despite a number of attempts. Currently, we are unable to progress further with the LaTeX manuscript conversion and therefore hope submitting in .doc file in the correct format is satisfactory given this included on the Plos One submission guidelines as an appropriate document type. If LaTeX submission is required, we will require further advice on how to do this, and further time extension.

We feel passionately that the manuscript is an important one for the Plos One readers and hope this edited and improved manuscript will be received well and look forward to your response.

Yours sincerely,

Dr Naina McCann

Corresponding author

Naina.mccann@nhs.net

We hope the revised manuscript is received well and look forward to hearing from you.

Yours sincerely,

Dr Naina McCann

Corresponding author

Naina.mccann@nhs.net
